# Late-life Disability May Increase with More Frequent Insomnia Symptoms and Sleep Medications Use Over Time

**DOI:** 10.1093/geroni/igaf122.3100

**Published:** 2025-12-31

**Authors:** Tuo Yu Chen, Soomi Lee, Orfeu Buxton

**Affiliations:** Academia Sinica, Taipei City, Taiwan (Republic of China); The Pennsylvania State University, University Park, Pennsylvania, United States; The Pennsylvania State University, University Park, Pennsylvania, United States

## Abstract

**Study Objectives:**

This study investigated whether disability status increased with the frequency of insomnia symptoms and sleep medication usage over a 5-year period and whether frequent use of sleep medication modified the longitudinal effects of insomnia symptoms on disability among community-dwelling older adults.

**Methods:**

Data were from the National Health and Aging Trends Study (2011-2015; n = 6,722). Disability was assessed with a validated disability index. Insomnia symptoms were assessed by the average frequency of longer sleep onset latency and trouble staying asleep. General sleep medication usage frequency was assessed. Multilevel modeling was used to analyze the data, considering health status, demographic information, and risky health behavior.

**Results:**

In the conditional growth model, for every one-unit higher in the frequency of insomnia symptoms and sleep medication usage, disability scores increased by 0.20 (SE = .02, p < .001) and 0.19 (SE = .02, p < .001) every year, respectively, adjusting for covariates. Moreover, the frequency of sleep medication usage influenced the relationship between insomnia symptoms and disability. Specifically, more frequent insomnia symptoms were associated with higher disability scores, and using sleep medications more often led to an even greater increase in disability scores than insomnia symptoms alone.

**Conclusions:**

Disability increased with more frequent insomnia symptoms and more frequent sleep medication usage each year. More frequent sleep medication usage has detrimental implications on disability beyond insomnia symptoms. Treating insomnia and managing sleep medication properly may help prevent disability among older adults.

